# Mesenchymal Stromal Cells and Viral Infection

**DOI:** 10.1155/2015/860950

**Published:** 2015-07-29

**Authors:** Maytawan Thanunchai, Suradej Hongeng, Arunee Thitithanyanont

**Affiliations:** ^1^Department of Pediatrics, Faculty of Medicine, Ramathibodi Hospital, Mahidol University, Bangkok 10400, Thailand; ^2^Department of Microbiology, Faculty of Science, Mahidol University, Bangkok 10400, Thailand

## Abstract

Mesenchymal Stromal Cells (MSCs) are a subset of nonhematopoietic adult stem cells, readily isolated from various tissues and easily culture-expanded *ex vivo*. Intensive studies of the immune modulation and tissue regeneration over the past few years have demonstrated the great potential of MSCs for the prevention and treatment of steroid-resistant acute graft-versus-host disease (GvHD), immune-related disorders, and viral diseases. In immunocompromised individuals, the immunomodulatory activities of MSCs have raised safety concerns regarding the greater risk of primary viral infection and viral reactivation, which is a major cause of mortality after allogeneic transplantation. Moreover, high susceptibilities of MSCs to viral infections *in vitro* could reflect the destructive outcomes that might impair the clinical efficacy of MSCs infusion. However, the interplay between MSCs and virus is like a double-edge sword, and it also provides beneficial effects such as allowing the proliferation and function of antiviral specific effector cells instead of suppressing them, serving as an ideal tool for study of viral pathogenesis, and protecting hosts against viral challenge by using the antimicrobial activity. Here, we therefore review favorable and unfavorable consequences of MSCs and virus interaction with the highlight of safety and efficacy for applying MSCs as cell therapy.

## 1. Introduction

Mesenchymal Stromal Cells (MSCs) are nonhematopoietic stem cells which have high proliferation, self-renewal, and multilineage differentiation capabilities. They are heterogeneous plastic-adherent cells that are initially expanded from bone marrow (BM) but can be isolated and culture-expanded from adipose tissue, fetal liver, placenta, and umbilical cord blood. MSCs can undergo differentiation into a variety of tissue types, including bone, cartilage, and muscle and still retain this multipotency after several rounds of expansion. MSCs isolated from most tissues commonly express CD105, CD73, and CD90 and lack expression of hematopoietic lineage markers including CD45, CD34, CD14 or CD11b, CD79a or CD19, and HLA-DR [1–6]. Advances in preclinical and clinical models of transplanted MSCs strongly support the potential role of MSCs on tissue regeneration and homeostasis [7, 8]. The major sources of MSCs which have been widely reported in clinical trials in terms of regenerative medicine are bone marrow, adipose tissues, and umbilical cord blood [9]; for example, (i) autologous bone marrow MSCs (BM-MSCs) transplantation could improve the short-term efficacy for the treatment of liver failure caused by hepatitis B and also the prognosis of liver function in end-stage liver disease [10, 11] and (ii) MSCs derived from adipose tissues (AD-MSCs) have been proven to be safe for using as therapeutic agents for autoimmune-mediated disorders, cardiovascular diseases, and soft tissue regeneration [12–14].

Numerous studies have shown that MSCs possess immunoregulatory properties by modulating the proliferation and function of several immune cells, for example, inhibiting differentiation of monocytes into dendritic cells (DCs), altering the cytokine profiles of DCs to result in an upregulation of regulatory cytokines and downregulation of inflammatory cytokines, inducing tolerant phenotypes of naive and effector T cells, inhibiting antibody production by B cells, and suppressing NK cell proliferation and NK cell-mediated cytotoxicity [15–19]. These immunomodulatory activities are mediated by both cell-cell interactions and secreted cytokines including interferon- (IFN-) *γ*, indoleamine 2,3-dioxygenase (IDO), transforming growth factor- (TGF-) *β*, interleukin (IL-) 6, IL-10, and prostaglandin E2 [20–23]. Given the immunomodulatory activity of MSCs, together with their low MHC class I expression, MSCs have been utilized to prevent and/or treat steroid-resistant graft-versus-host disease (GvHD) in patients undergoing allogeneic hematopoietic stem cell transplantation (HSCT) that have failed treatment with conventional immunosuppressant drugs. Administration of MSCs has successfully reduced the incidence and severity of GvHD and has also improved the outcomes of clinical diseases associated with aberrant immune responses [9, 24].

Although clinical applications of MSCs in cellular therapy have shown promising outcomes, viral reactivation, herpesvirus family especially still minimizes the transplantation efficacy and is associated with high morbidity and mortality rates in recipients. Viral reservoir in MSCs could be a causative agent of transplantation-related complications as it is able to increase the risk of viral transmission in recipients. Thus, prescreening of donors by using reliable and high efficient approaches is needed. In addition, high susceptibilities to several types of virus* in vitro* have raised safety concerns in applying MSCs for the treatment of virus-associated diseases [25–27]. However, there is limited data about the exact response of MSCs on viral infection in clinical settings. Virus and MSCs interaction may cause serious symptoms in immunocompromised individuals by virus-induced MSCs functional changes and MSCs-facilitated viral transmission to other tissues. Concurrently, however, this interaction also offers beneficial effects including the protection of the host from viral challenge by exertion of partial antiviral response in an infectious microenvironment. In this review, we present current information about benefits and drawbacks of MSCs upon encountering virus.

## 2. Safety in Using MSCs as Cellular Therapy in Virus-Related Complications

**Figure 1:**
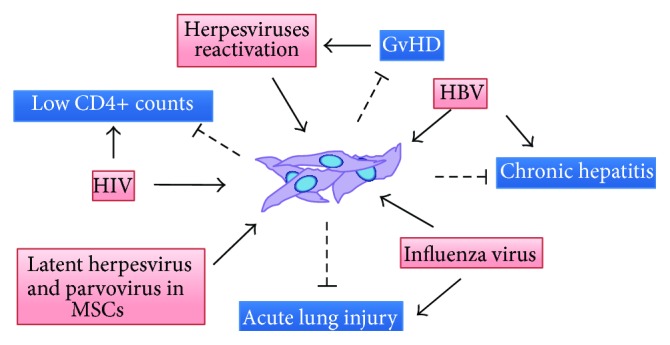
The proposed double-edge sword effect of using MSCs as a treatment for viral diseases. Several transplant-related complications and viral-associated diseases such as GvHD, low CD4+ numbers, ALI, and chronic hepatitis have been successfully improved by administered MSCs. The susceptibilities of MSCs to each viral infection may occur concurrently after infusion according to several infection evidences* in vitro*. GvHD, graft-versus-host disease; ALI, acute lung injury; HIV, Human Immunodeficiency Virus; HBV, Hepatitis B Virus; MSCs, Mesenchymal Stromal Cells.

In addition to GvHD prevention, MSCs become a promising tool for treatment of virus-associated diseases such as immunologic abnormality in Human Immunodeficiency Virus (HIV), chronic hepatitis in Hepatitis B Virus (HBV), and acute lung injury (ALI) in influenza virus. Administration of MSCs to virus-infected patients could impair the clinical efficacy if MSCs were targeted by viruses as they express receptors and coreceptors for the entry of several types of virus. Moreover, the incidence of viral reactivation has been reported in immunocompromised individuals. As there is no available data regarding direct viral infection to MSCs in transplanted patients, we therefore presented the regenerative abilities of MSCs in viral-associated diseases and possible susceptibilities to each virus after MSCs transplantation (Figure 1).

### 2.1. Herpesviruses and Parvovirus

Herpesviruses including cytomegalovirus (CMV), herpes simplex-1 (HSV-1) and herpes simplex-2 (HSV-2), Epstein-Barr Virus (EBV), and Varicella Zoster Virus (VZV) represent a prominent pathogen in immunocompromised hosts [28, 29]. MSCs have been shown to be susceptible to infection by these herpesviruses and become functionally defective following infection. Smirnov et al. demonstrated that CMV infection of MSCs could interfere with the expression of cell surface molecules which are important for MSC interaction with cells of hematopoietic lineage. Moreover, CMV also impaired the adipogenic and osteogenic differentiation processes [30]. A recent study revealed that CMV infection critically impaired both MSC-mediated immunosuppressive and microbial activities by affecting IDO expression, a positive regulator of these functions [31]. Since MSCs can be isolated from various tissues/organs such as liver, brain, lung, and BM, Soland et al. demonstrated that MSCs derived from these organs were susceptible to CMV infection and that the virus is able to establish a productive infection and propagate within these cells* in vitro* [32].

Other members of the herpesvirus family have been shown to infect MSCs* in vitro* as well; fetal membrane derived Mesenchymal Stromal Cells (FM-MSCs) were fully permissive to infection with HSV-1, HSV-2, and VZV but not EBV and human herpesviruses 6, 7, and 8 (HHV-6, HHV-7, and HHV-8). These viruses were capable of entering FM-MSCs but no productive infection occurred as viral gene expression was limited. However, the presence of herpesviruses genome in FM-MSCs should be screened since FM becomes an alternative source of MSCs for transplantation [33]. The evaluation of herpesviruses in MSC donors is such an important matter in terms of safety prior transplantation, as 7 out of 19 healthy HCMV-seropositive donors of BM-MSCs have been found to harbor low copy numbers of HCMV DNA, which potentially serve as HCMV reservoir in transplant patients [32]. Regarding viral reservoir and transmission issues, the presence of parvovirus B19 (B19) DNA in MSCs obtained from healthy donors revealed by two studies should also be focused [34, 35] since MSCs, which express B19 receptor, were permissive for B19 and could transmit virus to hematopoietic cells* in vitro* [34]. Thus, highly sensitive detection methods are needed to screen for the presence of viral DNA in MSCs in both donor and recipients in order to reduce the incidence of any viral-associated diseases and to assure the safety and efficacy of MSCs-based therapy.

### 2.2. Human Immunodeficiency Virus (HIV)

HIV-1 pathogenesis is characterized by the progressive depletion of CD4+ T cells, leading eventually to clinically significant immunodeficiency [36]. MSCs have been proposed to have the ability to improve host immune reconstitution outcomes in HIV-infected highly active antiretroviral therapy- (HAART-) treated nonimmune responders (NIRs) by acting to decrease the activation of CD8+ T cells which may lead to more effective CD4+ T cell restoration. MSC recipients showed a significant increase in both naïve and central memory CD4+ T cell counts and also in cytokine production in response to an HIV antigen [37]. Although transfused MSCs are well tolerated and safe for recipients, the susceptibility of MSCs to HIV-1 and the outcomes of this infection are an important matter of concern. HIV-1 infection of bone marrow stromal cells could suppress the clonogenic potential of MSCs and increase the levels of proinflammatory cytokines (TNF-*α*, IL-1*β*, IL-6, and MIP-1*α*), suggesting a possible role of HIV-1-associated bone marrow abnormalities [38]. Cotter et al. used an* ex vivo* experimental model to demonstrate that treatment of MSCs with serum from HIV-1 patients can alter MSC osteogenic and adipogenic differentiation [39]. However, the inhibitory effects of HIV-1 protease inhibitors (PIs) present in the serum samples on these differentiation processes cannot be ruled out, as it has been reported that treatment with PIs is associated with lipid metabolism disorders [39]. In addition, HIV-1 infection has been shown to be involved in the differentiation derangement of MSCs derived from the vascular wall. HIV-1 is able to integrate its genome into the DNA of MSCs in the vascular wall, suggesting that MSCs may act as a potential infection reservoir [40]. MSCs isolated from Tg26 HIV-1 transgenic mice displayed HIV genes. Proliferation, differentiation, and cytokine production of these MSCs were strikingly impaired. Moreover, transplantation of Tg26 HIV-1 MSCs improved outcomes less effectively compared with healthy MSCs in mice with acute renal injury [41]. Taken together, these data are representative of the situation when healthy MSCs are infused to HIV patients, and also when HIV-MSCs are given to transplant recipients. Either of these actions could undermine the clinical efficacy of transplantation.

### 2.3. Hepatitis B Virus (HBV)

Hepatitis B is one of the most common infectious diseases. There are about 360 million people who have a chronic HBV infection, and 0.6 million people die each year from HBV related liver disease or hepatocellular carcinoma (HCC) worldwide [42]. At present, the therapeutic treatments for chronic hepatitis B are limited. Orthotopic liver transplantation (OLT) remains the only therapeutic option for patient with end-stage liver disease caused by chronic HBV infection, but it is limited by the shortage of organ donors as well as the susceptibility of the transplanted tissue to reinfection by HBV [43]. Transplantation of MSCs is being considered as a candidate therapeutic approach for improving HBV related liver disease since MSCs can be induced into hepatocyte-like cells, which are capable of expressing a subset of hepatic genes and showing hepatic functions including glycogen production and albumin secretion [43, 44]. Initial findings revealed that human BM-MSCs were able to improve liver function in hepatitis B patients with end-stage liver disease [45]. However, the question remains as to whether infused MSCs are susceptible to HBV infection and this cell type acts as an extrahepatic virus reservoir. Xie et al. isolated MSCs from BM of hepatitis B patients and found that both BM-MSCs and BM-MSCs undergoing hepatocytes differentiation are resistant to HBV infection* in vitro* [46]. Conversely, BM-MSCs obtained from healthy donors fully supported HBV infection, replication, expression, and secretion, which could make the MSCs a reservoir of virus [42]. Furthermore, it has been reported that MSCs can serve as an extrahepatic virus reservoir by harboring and transporting HBV to the injured tissues after transplantation of HBV-exposed MSCs into myocardial infarction (MI) mouse model [47]. Although autologous BM-MSCs were resistant to HBV infection and were proven to be safe for transplantation, obtaining autologous BMSCs from chronic hepatitis B patients is too invasive and may cause distress to patients. In addition, it has been reported that BM-MSCs from chronic hepatitis B patients proliferate defectively and have a low expression level of growth factor receptors [48]. Thus, BM may not be a good source for MSC isolation for autologous transplantation of BM-MSCs. A recent study suggested an additional source of hepatic cells, adipose-derived MSCs (AD-MSCs). Wang et al. demonstrated that AD-MSCs could differentiate into functional hepatocyte-like cells. Interestingly, AD-MSCs as well as AD-MSCs undergoing hepatic differentiation were not susceptible to infection by HBV* in vitro*. AD-MSCs may thus be an ideal MSCs source for chronic hepatitis B patients [43]. However, further long-term monitoring of transplanted AD-MSC to HBV infection* in vivo* and in randomized clinical trials is required.

### 2.4. Avian Influenza Virus (AIV)

Avian influenza virus (AIV) causes disease among birds species, including chickens, ducks, and turkeys. There are several AIV subtypes such as H5N1, H7N9, and H9N2 that can cross species barriers and become infectious to mammals [49, 50]. Symptoms of avian influenza in humans have ranged from typical human influenza-like symptoms (e.g., fever, cough, sore throat, and muscle aches) to pneumonia, severe respiratory diseases (such as acute respiratory distress and acute lung injury), uncontrolled systemic inflammatory response, and other severe life-threatening complications [51, 52]. However, the symptoms of avian influenza may depend on which virus caused the infection [50]. Although antiviral drug can reduce the severity and duration of symptoms, it does not eliminate flu symptoms or repair the tissue injury caused by virus-associated inflammation. It has been suggested that anti-inflammatory therapies may attenuate viral-induced lung injury in mice [53]. Given that MSCs possess immunomodulatory and regenerative properties and capability to secrete endothelial and epithelial growth factors, Li et al. demonstrated that treatment with MSCs greatly improved acute lung injury induced by the H9N2 virus in mice [62], although recent studies have shown that administration of MSCs in a prophylactic or therapeutic regimen failed to alleviate the outcomes of acute severe influenza [63, 64]. However, MSCs have been shown to be susceptible to avian influenza virus infections. Our previous data revealed that human primary MSCs were permissive to highly pathogenic avian influenza A (H5N1) virus (HPAI H5N1) infection; the infection resulted in apoptosis and losing of the immunoregulatory activity of MSCs [65]. Likewise, Khatri et al. showed evidence supporting the replication of both the avian H1N1 and avian H9N5 influenza strains by primary chicken MSCs which resulted in cell lysis and cytokine and chemokine production [66]. However, there is no current evidence of direct infection of influenza viruses to infused MSCs* in vivo*. In addition, the conflicting observations of MSCs-mediated tissue regeneration may result from different virus strains and experimental designs. Thus, it is too soon to make a conclusion of this topic. More information regarding MSCs targeted by influenza viruses in both preclinical and clinical models are necessary for the field of translational medicine.

## 3. The Beneficial Effects of MSCs on Viral Infection

### 3.1. The Differential Effect of MSCs on T Cell Responses to Viral Infections

It is widely known that MSCs can suppress alloantigen-induced T cell functions* in vitro*. However, the immunosuppressive effect of MSCs on the immune response to infectious pathogens remains controversial. An antiviral response is crucial for viral eradication and for the prevention of the progression of virus-associated disease. Since this immunosuppressive effect is nonspecific, both alloantigen and viral antigen are able to be suppressed, and thus this may be detrimental in clinical settings in which viral exposure is common. In contrast, if antiviral specific T cells are allowed to function in the presence of MSCs, they are capable of maintaining host defense integrity against infections. Therefore, it is important to know how MSCs affect virus-specific T cell effector functions. Differential effects of MSCs on immune cells in response to viral infection or infectious agents were summarized in Table 1. Karlsson et al. found that MSCs have little inhibitory effect on the antiviral T cell response. EBV-specific cytotoxic T cells (CTLs) or CMV-CTLs cultured with MSCs retained the ability to proliferate and produce IFN-*γ* in response to viral antigens and to kill virus-infected cells* in vitro* [56]. MSCs-derived IFN-*γ* was believed to be responsible for offsetting the immunosuppressive effect of MSCs by mediating the partial CTL responses during viral infection [67]. The dual effects of MSCs on immune response were later confirmed by Li et al. in which IDO is responsible for switching an immune-modulator to immune-enhancer [68]. According to these dual effects, the drawbacks of immune regulatory action of MSCs in the infectious environment have been reported; for example, (i) Sundin et al. demonstrated that lymphocyte proliferation induced by CMV antigen was suppressed in the presence of MSCs [57], (ii) Malcherek et al. found that MSCs suppressed proliferation and the release of IFN-*γ* of CMV and influenza-specific T cells [58], and (iii) UC-MSCs were recently shown to inhibit the cytotoxicity of V*γ*9V*δ*2 T cells against H1N1 influenza virus* in vitro* [59], supporting the evidences of prolonged infection in recipients. Thus, this is important for medical specialists to be aware of dual effects of MSCs on immune system against viral infection in using MSCs as regenerative medicine.

In addition to* in vitro* studies, there have been clinical reports showing that two patients who received MSCs for acute GvHD had persistent CMV-specific T cells and retained IFN-*γ* response to CMV infection [56]. The other clinical studies also suggested that treatment with MSCs did not increase the risk of viral reactivation or impair the host immune response because children given MSCs did not get more viral infections compared to historic control groups [55, 69]. However, receiving MSCs has been shown to be one of the risk factors for VZV reactivation in VZV-seropositive patients undergoing allogeneic HSCT possibly due to MSCs-mediated immunomodulatory activity [61]. A recent publication has reported conflicting results between* in vitro* and clinical studies; HAdV-specific T cell activation was not affected by MSCs* in vitro*, whereas HAdV infection was associated with decreased survival in children treated with MSCs [54]. Due to controversial studies together with small number of studies related to viral infection after being treated with MSCs, the conclusion of this issue cannot be made at the moment, and future studies should address more solid evidences of differential effects of MSCs on immune response when using MSCs in clinical applications.

### 3.2. MSCs Can Be Used as a Tool for Investigating Viral Pathogenesis

To understand the pathogenicity of viruses, it is important to understand their natural life cycle. To date, there is no* in vitro* assay for HBV natural infectivity available; thus the early steps of the viral life cycle are not well understood. The hepatoma-derived cell lines, well established for* in vitro* study of HBV, are not suitable for studying the mechanism of the early stages of virus-host interactions, including viral attachment, penetration, and uncoating, because the viral genome is introduced by integration or transfection rather than by infection [70]. In addition, the use of primary human hepatocytes, which support natural penetration and full viral replication, is hampered by limited resources and the technical difficulties that are associated with the culture methods. Therefore, an ideal cell source for the study of HBV* in vitro* is needed. As mentioned in the dark side section, human BM-MSCs can differentiate into functional hepatocyte-like cells* in vitro* and restore liver function in animal models of liver failure [44, 71]. Interestingly, BM-MSCs fully supported the complete HBV life cycle, including uptake, entry, infection, replication, and production of infectious viral progeny with comparable efficiency with the infection of primary human hepatocytes and human hepatoma cell lines. The achievement of supportive viral proliferation without the loss of native viral characteristics is far superior to primary hepatocytes [42]. Although human BM-MSCs are susceptible to HBV infection which is classified into the drawbacks of MSCs in terms of cellular therapy, the infection data offers a new opportunity for basic research on the HBV life cycle and the mechanism that mediates the early stages of virus-cell interactions.

Another useful application of the mesenchymal precursor for viral pathogenesis was demonstrated in Kaposi's sarcoma-associated herpesvirus (KSHV), a cause of several malignancies in AIDS patients. For KSHV, the mechanism of virus-induced oncogenesis remains elusive due to the lack of a good experimental model for studying the cellular transformation of primary cells [72]. Jones et al. found that KSHV efficiently infects and transforms primary rat embryonic metanephric mesenchymal precursor (MM) cells, mesenchymal cells adjacent to the tips of the branching ureteric bud forming the nephrons. KSHV-transformed MM (KMM) cells presented spindle-shaped morphology, expressing vascular endothelial, lymphatic endothelial, and mesenchymal markers. KMM cells were immortalized, exhibited rapid proliferation, loss of contact inhibition in culture, and efficiently induced tumors when implanted in nude mice [73]. KSHV could, in fact, infect human mesenchymal cells but failed to immortalize and transform these cells [74]. Thus, it is important to note the limitations of cross-species observations in this study. In conclusion, this system could facilitate study of the factors as well as the mechanisms of KSHV-induced malignant transformation.

### 3.3. MSCs Possess Antiviral Effector Function

Since MSCs are attractive tools for the treatment of immune-mediated disorders including GvHD, a condition associated with a high risk of infection, the antimicrobial effector function of MSCs was studied in order to further evaluate the therapeutic potential of these cells in transplant patients. Interestingly, MSCs were capable of producing and secreting substantial quantities of the antimicrobial peptide, human cathelicidin hCAP-18/LL-37, which participated in bacterial clearance both* in vitro* and* in vivo* [75]. In addition, tryptophan-catabolizing enzyme IDO-positive human MSCs triggered by stimulation with inflammatory cytokines exhibited broad-spectrum antimicrobial effector function directed against a range of clinically relevant bacteria, protozoal parasites, and viruses [21]. However, there is limited data showing a potent antimicrobial activity against viruses, especially human herpesviruses, which is a prominent pathogen in the setting of allogeneic hematopoietic stem cell transplantation. Meisel et al. found that the decrease of CMV and HSV-1 replication in MSCs was mediated by IDO expression in the presence of IFN-*γ*. This effect was abrogated by adding the IDO inhibitor to the system, indicating that IDO-mediated tryptophan catabolism is critically involved with antiviral effector function of MSCs [76]. Due to limited available data, the beneficial effects of antiviral activity of MSCs cannot be concluded at this time. Different factors involved with antimicrobial activity, types of virus, and preclinical and clinical studies have to be future investigated to address the exact antimicrobial effector function of MSCs.

## 4. Conclusion and Future Directions

The interplay between MSC and virus can be defined as double-edge sword. Upon encountering virus, MSCs appear to produce deleterious effects and act as viral transmitters which may subsequently worsen a therapeutic efficacy. Thus, the safety of MSCs administration should be ensured by screening the presence of viruses in donor and recipients. Since the clinical experiences of MSCs and viral infection remain largely unknown, more future studies addressing the behavior of MSCs in infectious environment would better explain this story. In addition, exploring the innate recognition for DNA/RNA viruses in MSCs and preactivating these molecules before infusion may help trigger the innate response pathway to inhibit viral replication in MSCs. Still, the data of antimicrobial activity of MSCs is restricted, and more evidences regarding new inhibitor molecules and different types of virus should be further investigated. A better understanding of the interplay between MSCs and virus will apparently delineate the safety and efficacy of using MSCs as cell therapy for treating GvHD and several degenerative tissues.

## Figures and Tables

**Table 1:** Differential effects of MSCs on immune response against viral infection or infectious agents.

Effects of MSCs on immune reactions in response to viral infection	Outcomes	
	Preclinical	Clinical
No or little effect	(i) Stable proportion of CMV- and HAdV-specific effector T cells [54]	(i) No viral reactivation [55]
	(ii) Retaining the ability of EBV- and CMV-specific CTLs to proliferate and produce IFN-*γ* [56]	(ii) Persistent CMV-specific T cells and IFN-*γ* response to CMV infection [56]

Suppressing	(i) Poor lymphocyte proliferative responses [57] (ii) Proliferation and IFN-*γ* production of CMV and influenza-specific T cells which were inhibited [58] (iii) Cytotoxicity of V*γ*9V*δ*2 T cells against influenza virus H1N1 which was inhibited [59]	(i) Decreasing survival of children treated with MSCs due to HAdV infection [54] (ii) An opportunistic viral infection developed in 3 of 6 patients receiving MSCs [60] (iii) VZV reactivation in VZV-seropositive patients [61]

MSCs, Mesenchymal Stromal Cells; CMV, cytomegalovirus; HAdV, human adenovirus; EBV, Epstein-Barr Virus; CTLs, cytotoxic T lymphocytes; IFN, interferon.
